# Sustained Type 2 Diabetes Remission and Metabolic Health Through Intensive Lifestyle Intervention: A Case Report With a Nine-Year Follow-Up

**DOI:** 10.7759/cureus.110884

**Published:** 2026-06-15

**Authors:** Baby Sharma, Pramod Tripathi, Nidhi S Kadam, Malhar Ganla

**Affiliations:** 1 Department of Research, Freedom From Diabetes Research Foundation, Pune, IND; 2 Department of Medicine, Freedom From Diabetes Clinic, Pune, IND; 3 Department of Kinesiology and Exercise Science, Freedom From Diabetes Clinic, Pune, IND

**Keywords:** intensive lifestyle intervention, long-term follow-up, non-pharmacological treatment, obesity, stress management, type 2 diabetes remission, vegan diet

## Abstract

A 61-year-old man with newly diagnosed type 2 diabetes (T2D), obesity by Asian-specific body mass index (BMI) criteria (28.8 kg/m²), dyslipidemia, and hypertension enrolled in a three-month intensive lifestyle intervention (ILI) at the Freedom from Diabetes Clinic, India. The ILI comprised a whole-food, plant-based, vegan diet, daily structured exercise (aerobic, resistance, and yoga), and stress reduction through meditation and journaling. At the four-month assessment following completion of the three-month ILI, body weight decreased from 79 kg to 68.6 kg, and glycated hemoglobin (HbA1c) fell from 10.7% to 5.7% without the use of glucose-lowering medication. The lipid profile improved markedly (low-density lipoprotein: 141 → 107 mg/dL; triglycerides: 192 → 81 mg/dL; and high-density lipoprotein: 41 → 52 mg/dL). By eight months, the HbA1c level reached 5.5%, fulfilling the consensus criteria for T2D remission. The patient maintained remission for nine consecutive years, as confirmed annually by a 75 g oral glucose tolerance test (OGTT). The annual OGTT demonstrated normal glucose tolerance on all nine occasions. This case illustrates that sustained T2D remission and broad metabolic improvement are achievable through intensive lifestyle modifications alone, even in a patient aged 61 years at diagnosis. This reinforces the role of structured lifestyle interventions as a viable first-line strategy for newly diagnosed T2D.

## Introduction

Type 2 diabetes (T2D) is a major and growing global health burden driven by sedentary lifestyles, energy-dense dietary patterns, and rising rates of overweight and obesity [1,2]. Although pharmacological therapy remains central to glycemic management, accumulating evidence demonstrates that intensive lifestyle interventions (ILI) incorporating structured dietary modifications, regular physical activity, and behavioral stress management can achieve substantial glycemic improvements and, in appropriately selected patients, induce T2D remission [3,4]. These findings align with the 2022 American Diabetes Association (ADA) and European Association for the Study of Diabetes (EASD) Consensus Report and the American College of Lifestyle Medicine (ACLM) position statement, all of which endorse lifestyle intervention as a first-line strategy in the management of newly diagnosed T2D [4,5].

T2D remission was defined as maintaining glycated hemoglobin (HbA1c) levels below 6.5% (48 mmol/mol) without any glucose-lowering medications for at least three months post-intervention [6]. Despite growing interest in lifestyle-induced remission, evidence regarding its long-term sustainability remains limited. Most clinical trials and case reports have reported follow-up periods of one to two years, with few documenting pharmacologically unsupported remission beyond five years [5,7]. Emerging evidence suggests that several baseline characteristics may predict successful long-term remission, including shorter diabetes duration, drug-naïve status at diagnosis, greater initial weight loss, and preserved beta-cell function. Whole-food, plant-based, vegan dietary patterns, particularly when integrated into comprehensive lifestyle interventions, have demonstrated significant cardiometabolic benefits in randomized controlled trials [8]. In the present case, this dietary approach was adapted to the South Asian cultural context and implemented as the central component of a multimodal ILI.

We report a case of a 61-year-old drug-naïve man with newly diagnosed T2D, obesity by Asian-specific criteria (body mass index (BMI) 28.8 kg/m²), dyslipidemia, and hypertension who achieved T2D remission following a three-month multimodal ILI comprising a whole-food plant-based vegan diet, structured aerobic and resistance exercise, yoga-based physical activity, and mindfulness-based stress management, without pharmacological intervention. To our knowledge, this case represents one of the longest documented periods of lifestyle-induced T2D remission in the published literature, with normoglycemia confirmed by nine consecutive annual oral glucose tolerance tests (OGTT) over a nine-year follow-up period.

## Case presentation

A 61-year-old man, 165.5 cm (5 ft 5 in) in height and weighing 79 kg (174.16 lb) (BMI 28.8 kg/m², classified as obese), enrolled in a three-month diabetes management program at the Freedom from Diabetes Clinic, India, in July 2016. He had a graduate-level education and was employed in a sedentary, desk-based, salaried position. The patient was diagnosed with T2D during a random health checkup in June 2016. He joined the program in July 2016 and was drug-naïve (not on any glucose-lowering therapy) at the time of enrollment. He reported a maternal family history of diabetes and significant personal stress preceding the diagnosis. He also had a 1.8-year history of hypertension and was taking one tablet of antihypertensive medication (telmisartan 40 mg + amlodipine 5 mg) every day. He followed a predominantly vegetarian diet and occasionally consumed restaurant or outside food once a week. He abstained from alcohol, chewing tobacco, and smoking. The patient described significant occupational stress preceding the diagnosis and reported sleep disturbances characterized by difficulty in initiating sleep and frequent nocturnal awakenings. His demanding work environment was identified as a major psychosocial stressor before diagnosis. The patient did not report the use of Ayurvedic, herbal, complementary, or alternative therapies during the intervention or throughout the nine-year follow-up. Apart from the prescribed antihypertensive medications, the only additional supplements used were cholecalciferol (vitamin D) and vitamin B12, as detailed in Table 1.

**Table 1:** Changes in biochemical parameters over 9 y M: months; Y: years; HbA1c: glycated hemoglobin; FBG: fasting blood glucose; PP2: postprandial blood glucose (2 hours after eating); TC: total cholesterol; HDL: high-density lipoprotein; TG: triglycerides; LDL: low-density lipoprotein; VLDL: very-low-density lipoprotein; TC/HDL: total cholesterol/high-density lipoprotein ratio; LDL/HDL: low-density lipoprotein/high-density lipoprotein ratio; sCr: serum creatinine; HOMA-IR: Homeostatic Model Assessment of Insulin Resistance; hs-CRP: high-sensitivity C-reactive protein; ND: no data

	Parameters	Unit	Normal reference range	At enrollment	4 M	8 M	1 Y	3 Y	5 Y	6 Y	7 Y	9 Y	
													Glycemic	HbA1c	%	<6.5	10.7	5.7	5.5	5.5	5.6	5.5	5.9	6.1	6		
FBG	mg/dL	70-100	134	105	100	91	96	97	91	90	89			
PP2	mg/dL	<140	278	123	107	95	146	117	77	136	138			
Insulin fasting	µU/mL	2.6-24.9	9.3	9.8	4.4	7.2	6.9	8.36	8.32	7.56	5.1			
Lipid profile	TC	mg/dL	<200	220	175	197	176	219	221	218	208	206		
	HDL	mg/dL	>40	41	52	58	50	62	64	57	55	58		
	TG	mg/dL	<150	192	81	73	67	79	86	96	90	96		
	LDL	mg/dL	<100	141	107	124	113	141.2	139.8	141.8	135	128.8		
	VLDL	mg/dL	2-30	38	16	15	13	15.8	17.2	19.2	18	19.2		
	TC/HDL	-	≤5.0	5.37	3.37	3.4	3.52	3.53	3.45	3.82	3.78	3.55		
	LDL/HDL	-	≤3.6	3.44	2.06	2.14	2.25	2.28	2.18	2.49	2.45	2.2		
Kidney function	Blood urea nitrogen	mg/dL	5-20	7.9	11.2	9.3	9.35	7.5	10.3	8.5	11.12	9.9		
	sCr	mg/dL	0.7-1.3	0.8	0.7	0.8	0.85	0.89	0.84	0.78	0.93	0.92		
	Urine microalbumin	µg/mL	≤30	5.7	3.4	1.7	ND	0.05	2.5	5.5	2.8	10.1		
Others	Hemoglobin	g/dL	13.8-17.2	14.1	14.7	14.5	14.5	14.4	13.2	13.8	13.4	13.6		
	HOMA-IR	-	<2	3.08	2.54	1.09	ND	ND	ND	ND	ND	ND		
	Vitamin D	ng/mL	>20	60.4	69.5	64.2	63.6	42	41.9	43.1	42.2	57		
	Vitamin B12	pg/mL	200-900	1,616	653	1,017	879	787	1,115	803	863	625		
	hs-CRP	mg/L	<1	4.1	7.2	10	0.8	3.14	0.87	1.07	1.08	0.71		

Diagnostic assessment

At the time of enrollment, initial anthropometric and biochemical assessments revealed obesity, with a BMI of 28.8 kg/m². His HbA1c level was significantly elevated to 10.7% (96 mmol/mol), indicating suboptimal glycemic control in the absence of glucose-lowering medication. His fasting blood glucose level was 134 mg/dL, and his postprandial glucose level was markedly high at 278 mg/dL. The patient was also diagnosed with dyslipidemia at the time of enrollment, characterized by elevated total cholesterol (TC: 220 mg/dL), high low-density lipoprotein (LDL: 141 mg/dL), low high-density lipoprotein (HDL: 41 mg/dL), and increased triglycerides (TG: 192 mg/dL). Blood pressure readings were elevated at 146/80 mmHg, necessitating the continued use of antihypertensive medication. Insulin resistance was high based on the Homeostasis Model Assessment of Insulin Resistance (HOMA-IR) [9], which was 3.08 (reference range: <2.0).

Treatment

The patient completed a three-month ILI program, including a vegan diet and exercise regimen tailored and monitored by a physician and a mentor, respectively. The mentor was a former participant of the program who served as a “buddy” to new participants to help them navigate the intervention. The ILI also featured monthly group therapy sessions with meditation and journaling for stress management, aiming to improve overall health and manage the patient's condition.

Before the program, the patient followed a vegetarian diet, including milk and milk products. During the program, the dietary intervention excluded dairy products. A vegan diet was implemented, with an emphasis on meal timing and portion control. The patient's day commenced at 6 AM with a nutrient-rich smoothie containing leached leafy greens (spinach/other local greens), one fruit (apple/guava/banana/pear or papaya), two herbs (mint/basil/curry leaves/betel leaf), and a pinch of black pepper, turmeric powder, rock salt, lemon juice, and cinnamon powder consumed on an empty stomach for detoxification. For breakfast, the patient had raw food items such as sprouts (50%) along with cooked food made from lentils/pulses (remaining 50%) with no cereals or carbohydrates. Lunch and dinner were at 1 PM and 7:30 PM, respectively, and included only one grain in the meal with cooked vegetables, raw salad (whole/sprouted legumes and raw vegetables), and pulses/lentils in equal proportions (25% each). Additionally, the intake of soaked nuts and seeds was recommended as an evening snack (two tablespoons mixed with seeds of sunflower, pumpkin, watermelon, sesame, and flaxseed with 5-6 soaked almonds and two whole walnuts). The diet plan restricted the total calorie intake to 1,400-1,600 kcal/day. One month into the intervention, juice fasting [10] was recommended to accelerate weight loss. Juice fasting consisted of the consumption of green smoothies and vegetable juices once a week throughout the day to support initial weight loss and dietary transition. Fresh vegetable juices containing antioxidants and enzymes promote metabolic health and satiety while maintaining minimal calories.

The patient was spending 20-25 minutes swimming in the morning and 40-45 minutes in the gym in the evening daily, six days a week, at the time of joining the program. Additionally, the patient was prescribed a daily 35- to 40-minute exercise routine, including warm-up exercises, light-resistance training, and yoga-based cleansing exercises to improve lymphatic circulation and muscle strength. Surya Namaskar (sun salutation) was included to enhance flexibility and mobility. A 20-minute walk or walk-jog was recommended to improve cardiovascular health. Post-meal antigravity exercises [11] were recommended for 5-10 minutes to manage postprandial glucose spikes.

The patient was advised to meditate daily, focusing on mindfulness through deep breathing and positive affirmations to enhance their mental clarity and emotional balance. The SAAF GOAL method (S: scene, A: actors, A: actions, and F: feelings) [12] was introduced in the second month to help the patient clearly visualize goals, involving the scene, key actors, intended actions, and associated feelings. Additionally, regular journal writing was recommended as a stress-relief tool, aiding self-reflection and emotional processing. Individual calls with the mentor were also provided to relieve stress, achieve emotional stability, and monitor adherence.

Additional monthly educational sessions were conducted to improve patients’ understanding of self-management and reinforce lifestyle modification strategies. Throughout the intervention, the patient self-reported daily blood glucose measurements and body weight via WhatsApp (Meta Platforms, Inc., Menlo Park, CA, USA) to the assigned physician, who regularly monitored these data. These records were reviewed to monitor adherence to the prescribed lifestyle program and provide timely feedback when deviations from dietary or exercise recommendations were identified. Adherence was assessed through daily WhatsApp self-reporting of blood glucose and weight, supplemented by regular follow-up with the mentor. Additionally, the patient submitted photographs of meals via WhatsApp to the assigned physician, enabling real-time dietary review and targeted feedback on food choices throughout the three-month intervention period. Overall compliance with the dietary and exercise components of the program was high throughout the three-month intervention period. Given the patient's baseline history of occupational stress and sleep disturbances, the stress management component of the ILI included daily meditation, deep-breathing exercises, and reflective journaling. However, no formal assessment of sleep quality or sleep disorders, such as polysomnography or validated sleep questionnaires, was performed at the baseline or during follow-up.

Outcome and follow-up

At the four-month assessment following the completion of the three-month ILI, the patient showed notable health improvements. Tables 1, 2 show the changes in anthropometric measurements and biochemical parameters, respectively, tracked over nine years. His weight decreased from 79 to 68.6 kg, and his BMI decreased from 28.8 to 25 kg/m². His HbA1c levels also decreased from 10.7% to 5.7%, indicating substantial glycemic improvement without the use of glucose-lowering medications. The patient continued to adhere to the protocol beyond the intervention period. By the end of eight months, his weight had further reduced to 64.8 kg, and his BMI reached 23.6 kg/m². Notably, his HbA1c level declined to 5.5%, meeting the criteria for T2D remission. The patient's lipid profile showed significant improvement by eight months, with LDL cholesterol decreasing from 141 to 124 mg/dL, TG decreasing from 192 to 73 mg/dL, and HDL cholesterol increasing from 41 to 58 mg/dL. Despite improvements in metabolic parameters, blood pressure deteriorated, increasing from 146/80 mmHg at baseline to 162/92 mmHg at eight months, indicating the need for continued cardiovascular risk management. Based on biochemical investigations of vitamins (D and B12), the patient was advised to take a monthly cholecalciferol tablet to maintain vitamin D levels throughout the intervention. Additional nutritional supplements, like multivitamins (B12), were prescribed based on routine biochemical analyses (Table 1). Antihypertensive medications were continued. Insulin resistance improved markedly over the course of the intervention, with HOMA-IR decreasing from 3.08 at enrollment to 2.54 at four months and 1.09 at eight months (reference range: <2.0), reflecting a substantial restoration of insulin sensitivity. Formal sleep assessments were not conducted; however, the patient subjectively reported an improvement in sleep quality following the introduction of daily meditation and stress management practices. No validated psychological well-being scores (e.g., PHQ-9 for depression or GAD-7 for anxiety) were collected during the follow-up period; however, the patient reported reduced perceived stress and improved emotional stability, attributed to the meditation, journaling, and mentorship components of the ILI.

**Table 2:** Changes in anthropometric parameters over 9 years BMI: body mass index

Parameters		Unit	Normal reference range	At enrollment	4 months	8 months	9 years
Anthropometric	Height	cm	-	165.5	165.5	165.5	165.5
	Weight	kg	-	79	68.6	64.8	65.3
	BMI	kg/m²	18.5-24.9	28.8	25	23.6	23.8
	Waist circumference	cm	<90	97	92	88	86
Body composition	Waist-to-height ratio	-	<0.5	0.59	0.56	0.53	0.52
	Fat percent	%	10-20	32.9	30	24.1	26.8
	Visceral fat percent	%	≤10	16	12	9.5	11
	Subcutaneous fat percent	%	12-18	22.7	20.5	16.6	18.3
	Skeletal muscle mass	%	32-40	26.7	27.8	29.9	28.3
Blood pressure	Systolic	mmHg	90-120	146	152	162	162
	Diastolic	mmHg	60-80	80	87	92	82

**Figure 1:**
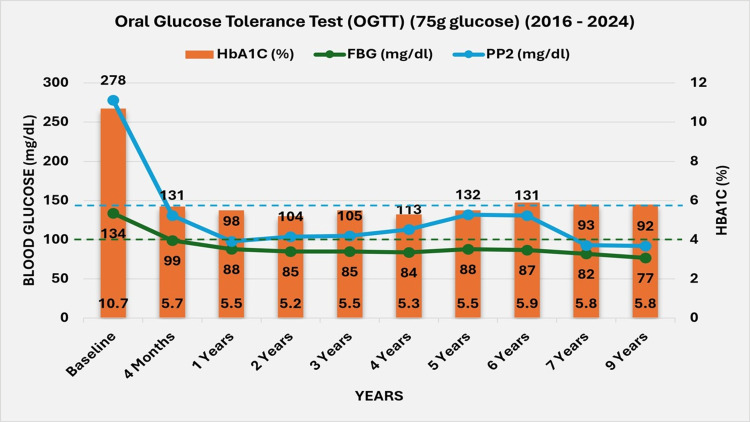
Yearly OGTT data Diagnostic reference thresholds are indicated as horizontal reference lines: FBG normal (<100 mg/dL) and PP2 normal (<140 mg/dL), per standard ADA criteria. HbA1c: glycated hemoglobin; FBG: fasting blood glucose; PP2: postprandial blood glucose (2 hours after eating); ADA: American Diabetes Association

The patient completed the three-month intervention in October 2016 and was subsequently followed up for nine years. Following program completion, he voluntarily continued to adhere to the prescribed dietary and exercise recommendations on most days and periodically sought clinical consultation to support the long-term maintenance of lifestyle changes. Formal follow-up assessments, including biochemical investigations, were conducted annually. No structured post-intervention support program was mandated; however, the patient maintained informal contact with the program’s mentorship framework and sought clinical guidance as needed during the follow-up. At the time of reporting, the patient had maintained substantial improvements in body weight, BMI, and glycemic control without using glucose-lowering medications. A modest increase in HbA1c was observed at the six-year follow-up (5.9%); however, the fasting blood glucose level remained within the normal range (87 mg/dL), and pharmacological treatment was not required. HbA1c remained below the diagnostic threshold for diabetes throughout the nine-year follow-up period. This is consistent with sustained T2D remission. The OGTT is considered the gold standard for diagnosing T2D because it is effective in evaluating how well a patient processes glucose [13]. The patient was advised to undergo a 75 g OGTT yearly. Figure 1 illustrates the results of annual OGTTs conducted between 2016 and 2024. The patient consistently cleared the OGTT every year (nine times), confirming sustained diabetes remission. Due to the high-stress working environment, the patient continued to take antihypertensive medication (telmisartan 40 mg + amlodipine 5 mg), half a tablet per day.

The data in Tables 1, 2 highlight the significant improvements in metabolic parameters achieved within the initial eight months, whereas Figure 1 confirms the long-term sustainability of these benefits through consistent annual OGTT clearance. Although cardiovascular parameters, such as blood pressure, showed an increasing trend, glycemic and lipid control remained stable, emphasizing the need for continued monitoring and management of cardiovascular health.

## Discussion

This case documents one of the longest published follow-ups of pharmacologically unsupported T2D remission achieved through ILI alone. A 61-year-old drug-naïve man with newly diagnosed T2D (HbA1c 10.7%), obesity status by Asian-specific criteria (BMI 28.8 kg/m²), dyslipidemia, and hypertension achieved sustained remission defined as HbA1c < 6.5% without glucose-lowering medication for at least three months within eight months of commencing a structured multimodal ILI and maintained that remission for nine consecutive years, confirmed by annual 75 g OGTT [6]. This time horizon substantially exceeds the two-year follow-up windows of landmark trials such as DiRECT and the LOOK AHEAD study, and no comparable case in the accessible peer-reviewed literature documents nine years of OGTT-confirmed remission without medication in a patient of this age. Therefore, this report fills an important evidence gap and reinforces the biological feasibility of durable T2D remission through sustained lifestyle adherence.

The glycemic improvement observed in this case was both rapid and sustained. HbA1c decreased from 10.7% at baseline to 5.7% within four months and further to 5.5% by eight months, without glucose-lowering pharmacotherapy. Fasting blood glucose remained between 75 and 89 mg/dL over nine years, while annual OGTT consistently demonstrated two-hour postprandial glucose levels below 140 mg/dL, confirming durable normoglycemia. The early intervention phase combined a calorie-restricted whole-food plant-based vegan diet with intensive daily physical activity, likely promoting the reduction of hepatic and pancreatic ectopic fat and restoration of insulin sensitivity. This is supported by the decline in HOMA-IR from 3.08 at baseline to 1.09 at eight months, suggesting the recovery of hepatic and peripheral insulin responsiveness. Similar mechanistic pathways have been described in the DiRECT trial, in which reductions in intrapancreatic fat were associated with restoration of first-phase insulin secretion and remission of T2D [5]. However, unlike DiRECT, where remission rates decline over time, the present case maintained normoglycemia without pharmacotherapy for nine years, indicating exceptional durability. The structured whole-food plant-based vegan dietary pattern is consistent with the findings from trials by Barnard et al., which demonstrated significant reductions in HbA1c and cardiometabolic risk factors in individuals with T2D following low-fat vegan interventions [14]. The present case extends these observations to a culturally adapted South Asian dietary framework. Weekly juice fasting during the intensive phase may further enhance caloric restriction, although evidence regarding its independent metabolic contribution remains limited.

The patient achieved a 14.2 kg weight reduction (17.9% of initial body weight) over eight months, with BMI normalizing from 28.8 kg/m² (obesity) to 23.6 kg/m² (normal). This reduction in body weight likely played a pivotal role in restoring insulin sensitivity and reducing insulin resistance [14]. Studies have consistently shown that a weight loss of 10%-15% can significantly improve glycemic control and potentially induce remission in individuals with T2D [5]. More informative than weight alone is the body composition trajectory: the visceral fat percentage fell from 16% (markedly elevated) to 9.5% at eight months and stabilized at 11% at the nine-year mark, remaining within or near the normal range throughout. Reduction of visceral adiposity is mechanistically linked to decreased hepatic lipid flux, reduced portal free fatty acid delivery, and consequent improvement in hepatic insulin sensitivity, the primary driver of fasting hyperglycemia in T2D [15]. The parallel improvement in HOMA-IR (3.08 → 1.09 at eight months) corroborates this pathway. Furthermore, maintenance of a healthy BMI throughout the follow-up period supports the association between sustained weight management and long-term remission [16].

ILI also led to significant improvements in the patients’ lipid profiles. The LDL cholesterol level decreased from 141 to 124 mg/dL by eight months, the TG level decreased from 192 to 73 mg/dL, and the HDL cholesterol level increased from 41 to 58 mg/dL. These improvements suggest a lower risk of cardiovascular complications, which is a critical consideration in patients with T2D [17]. However, despite improvements in the lipid profile, the patient’s systolic and diastolic blood pressure increased over time, underscoring the need for continued cardiovascular monitoring, even in the context of sustained glycemic remission. Concurrently, TC exceeded 200 mg/dL at the three-, five-, and six-year assessments (219, 221, and 218 mg/dL, respectively), further reinforcing the case for a formal cardiovascular risk review and targeted pharmacological intervention during the maintenance phase.

A noteworthy observation was the transient increase in high-sensitivity C-reactive protein (hs-CRP) during the initial months of intervention, rising from 4.1 mg/L at baseline to 7.2 mg/L at four months and 10.0 mg/L at eight months before normalizing to 0.8 mg/L by the end of the first year. This pattern is consistent with exercise-induced acute-phase response: sustained high-intensity physical activity, particularly the swimming and gym regimen undertaken in this case, is well recognized to transiently elevate inflammatory markers before inducing longer-term anti-inflammatory adaptations [18,19]. Intercurrent illness during the intensive phase could not be excluded; however, the subsequent sustained normalization of hs-CRP to well below 1 mg/L from year one onward supports a benign, exercise-related etiology rather than a pathological inflammatory process. Long-term adherence to dietary and physical activity regimens is often challenging for individuals undergoing lifestyle interventions [20,21]. However, this patient exhibited remarkable compliance with the prescribed vegan diet, routine exercise, and stress management practices over nine years. His ability to sustain these changes highlights the importance of patient motivation, behavioral counseling, and continuous monitoring to reinforce adherence to lifestyle changes [20]. Studies have shown that long-term lifestyle changes are more likely to be maintained when patients are provided with ongoing support, education, and periodic follow-ups [20,21].

The inclusion of stress management techniques, such as meditation, journaling, and mindfulness, likely contributed to improved psychological well-being and reduced stress-related hyperglycemia [12,22,23]. Stress plays a pivotal role in glycemic control by increasing cortisol secretion, thereby exacerbating insulin resistance [23,24]. The patient’s consistent engagement in stress management practices helped mitigate these effects, thereby supporting glycemic stability.

Limitations and clinical interpretation

This report is limited by its single-patient design, which precludes causal inference and limits its generalizability. The intervention required substantial patient engagement and included intensive exercise, structured dietary modification, stress management practices, and mentorship support, potentially limiting its scalability in routine clinical settings. In addition, formal psychosocial assessments, longitudinal measures of insulin resistance, such as HOMA-IR, and imaging-based quantification of ectopic fat were not performed. Selection bias is an important consideration. The patient voluntarily enrolled in an intensive clinic-based program and may represent a highly motivated subgroup that is not reflective of the broader T2D population. His graduate-level education, stable salaried employment, and access to specialist care may have independently facilitated long-term adherence and may not be broadly generalizable to other patients. Furthermore, the sustained adherence required for this intervention, including a whole-food plant-based vegan diet, daily structured exercise, and ongoing stress management practices over nine years, may not be feasible for many individuals without substantial behavioral support. Adherence assessment relied primarily on self-reported blood glucose and weight monitoring via WhatsApp, supplemented by regular mentor follow-ups. Objective adherence measures, such as validated dietary assessments, accelerometry, and biochemical dietary biomarkers, were not available. Although nine consecutive annual OGTTs and stable long-term metabolic outcomes provide indirect objective evidence of sustained lifestyle adherence, the absence of a standardized post-intervention follow-up protocol limits its reproducibility. Despite these limitations, the sustained nine-year remission of T2D without glucose-lowering medication, supported by serial OGTTs, persistent glycemic control, and the absence of diabetes-related complications, suggests that durable remission may be achievable in selected individuals through ILI. While findings from a single case should be interpreted cautiously, this report highlights the potential long-term durability of multimodal lifestyle approaches and supports the need for prospective studies to evaluate scalable strategies for sustained diabetes remission across diverse patient populations.

## Conclusions

This case demonstrates that sustained T2D remission, confirmed annually by a 75 g OGTT over nine consecutive years, is biologically achievable through a structured, multimodal ILI alone, without pharmacological glucose-lowering therapy. The patient's near-complete HbA1c normalization (from 10.7% to a sustained 5.5%-6.1% range), resolution of insulin resistance (HOMA-IR from 3.08 to 1.09), and absence of diabetic microvascular complications over nine years of follow-up challenge the prevailing clinical assumption that T2D is inherently progressive. The multimodal nature of ILI, integrating a culturally adapted whole-food plant-based vegan diet, structured aerobic and resistance exercise, yoga, peer mentorship, digital self-monitoring, and mindfulness-based stress reduction, likely underpins the exceptional durability of remission observed in this case. Unresolved challenges, including persistent hypertension and LDL cholesterol rebound from year three onward, highlight that lifestyle intervention does not eliminate the need for cardiovascular risk monitoring and targeted pharmacotherapy where indicated. This case supports the adoption of ILI as a viable, evidence-aligned first-line strategy for newly diagnosed T2D and provides motivation for prospective trials examining its long-term efficacy across diverse patient populations and clinical settings.
